# Ambient Aqueous Growth of Cu_2_Te Nanostructures with Excellent Electrocatalytic Activity toward Sulfide Redox Shuttles

**DOI:** 10.1002/advs.201500350

**Published:** 2016-02-03

**Authors:** Chao Han, Yang Bai, Qiao Sun, Shaohua Zhang, Zhen Li, Lianzhou Wang, Shixue Dou

**Affiliations:** ^1^ Institute for Superconducting and Electronic MaterialsAustralian Institute for Innovative MaterialsUniversity of WollongongSquires WayNorth WollongongNSW2500Australia; ^2^ Nanomaterials CentreSchool of Chemical Engineering and Australian Institute for Bioengineering and NanotechnologyThe University of QueenslandBrisbaneQLD4072Australia; ^3^ School of Radiation Medicine and Radiation ProtectionCollaborative Innovation Center of Radiation Medicine of Jiangsu Higher Education InstitutionsSoochow University199 Ren Ai Road, Suzhou Industrial ParkSuzhou215123P.R. China

**Keywords:** anion exchange, copper chalcogenides, electrocatalyst, surfactant‐free

## Abstract

A new aqueous and scalable strategy to synthesize surfactant‐free Cu_2_Te nanotubes and nanosheets at room temperature has been developed. In aqueous solution, Cu_2_E (E = O, S, Se) nanoparticles can be easily transformed into Cu_2_Te nanosheets and nanotubes via a simple anion exchange reaction under ambient conditions. The formation of Cu_2_Te nanosheets is ascribed to a novel exchange‐peeling growth mechanism instead of simple Kirkendall effect; and the resultant nanosheets can be further rolled into nanotubes with assistance of stirring. The morphologies of Cu_2_Te nanosheets and nanotubes can be easily controlled by changing the synthesis parameters, such as the concentration of precursors, the size of nanoparticle precursor, and the amount of NaBH_4_, as well as the stirring speed. Thus‐formed Cu_2_Te nanostructures exhibit excellent catalytic activity toward sulfide redox shuttles and are exploited as counter electrodes catalysts for quantum dot sensitized solar cells. The performance of Cu_2_Te nanostructures strongly depends on their morphology, and the solar cells made with counter electrodes from Cu_2_Te nanosheets show the maximum power conversion efficiency of 5.35%.

## 1 Introduction

As an important type of copper chalcogenides, copper telluride nanostructures, with a direct band gap between 1.1 and 1.5 eV, are conventionally used as back conductive contact materials for high‐efficiency CdTe based solar cells.^1^, ^2^, ^3^, ^4^, ^5^ Similar to other copper chalcogenides, the copper deficiency in Cu_2−_ *_x_* Te leads to a unique localized surface plasmon resonance (LSPR) in the near infrared (NIR) area, which makes it very attractive for surface‐enhanced Raman scattering (SERS) and photoacoustic imaging.^6^, ^7^, ^8^, ^9^, ^10^, ^11^ In addition, copper tellurides also are promising for potential applications in lithium ion batteries, photo‐thermal therapy, detection of toxic CO, and thermoelectric applications, 8, 10, 12, 13, 14 although these applications are strongly obstructed by the difficulty in controlled synthesis of their nanostructures with defined morphology, crystal structure, and composition.

In virtue of the complexity of tellurides and the inert property of Te element, the synthesis of nanostructured copper tellurides is more difficult than commonly reported prototypical semiconductors and usually needs harsh reaction conditions, such as high temperature, high pressure, or the usage of toxic organics.^6^, ^7^, ^8^, ^9^, ^10^, ^11^, ^12^ For example, cube‐, plate‐, and rod‐like copper telluride nanomaterials were prepared under the assistance of expensive LiN(Si(CH_3_)_3_)_2_ by the hot injection method.^8^ Copper telluride nanocubes, nanosheets, and hollow nanoparticles were synthesized by Han et al.^12^ in organic solvent at temperatures above 200 °C. These methods appeared to be either time or energy consuming, not to mention their low yield.

Herein, the highlight of this article lies in two aspects. First, we report a robust and scalable synthesis of surfactant free Cu_2_Te nanosheets and Cu_2_Te nanotubes from Cu_2_E (E = O, S, Se) nanoparticles via a simple anion exchange route at room temperature in aqueous solution. In principle, the anion exchange is similar to cation exchange based on the solubility difference of products. However, anion exchange is much more complex, because anion diffusion usually requires longer reaction times and higher temperatures than cation exchange due to the larger size of anions. In addition, changes in the morphology often accompany anion exchange reactions as anions usually form the framework of a structure.^15^, ^16^, ^17^, ^18^, ^19^ All these features make anion exchange more challenging than cation exchange to control both composition and morphology.^20^, ^21^, ^22^, ^23^, ^24^ However, detailed investigations on the mechanisms behind morphology change during the anion exchange reaction are rare, and such changes have been simply ascribed to the Kirkendall effect.^15^, ^16^, ^17^, ^18^, ^19^, ^21^, ^23^ In this article, we propose a novel exchange‐peeling mechanism for anion exchange reaction instead of simple Kirkendall effect, and the effect of different reaction parameters on morphologies of final product were thoroughly investigated.

Second, the morphology‐dependence catalytic reactivity of Cu_2_Te nanostructures has been observed for the first time. A potential application of Cu_2_Te nanostructures is its excellent electro‐catalytic property toward sulfide redox couples for use as counter electrodes (CEs) of quantum dot sensitized solar cells (QDSSCs). As an important member of QDSSCs, CdS, and CdSe cosensitized solar cells have attracted considerable attention, not only because the semiconductor quantum dot (QD) possess extraordinary light harvesting ability due to their tunable band gaps, but also because the inorganic nature of QD absorbers endow the device with good stability.^25^, ^26^, ^27^, ^28^, ^29^, ^30^, ^31^, ^32^ The CE of solar cells is an important part of the external circuit, and plays a pivotal role in catalyzing the redox reaction in the electrolyte, which is usually polysulfide electrolyte for CdS and CdSe cosensitized solar cells.^33^, ^34^, ^35^, ^36^, ^37^, ^38^ Most noble metals, such as Pt, show poor performance in polysulfide electrolyte because their surfaces are passivated by actively adsorbing sulfur atoms to result in lower conductivity, and considerable over‐potential is generated to slow electrolyte reduction.^33^, ^37^ Other alternatives include carbon materials and metal chalcogenides. Nevertheless, carbon materials, such as carbon black, graphene, and carbon nanotubes have disadvantages; carbon black lacks thickness for effective charge conduction although it possess large surface area, while graphene has poor catalytic activity even though it has high charge conduction.^36^ Metal chalcogenides, such as metal sulfides and selenides (e.g., Cu_2_S and Cu_2_Se), are found to show high power conversion efficiency (PCE).^33^, ^36^, ^38^ For example, by employing Cu_2_S as CE and ZnS/SiO_2_ coated CdSeTe as photo‐anode, the maximum power conversion efficiency (PCE) of Cd chalcogenide QDSSCs was boosted to 8.21%.^33^ In this paper, the performance of a new family of materials‐nanostructured Cu_2_Te with different morphologies as counter electrodes in CdS/CdSe cosensitized solar cells has been investigated. In comparison with conventional metallic CEs, our Cu_2_Te based nanostructures exhibit high electrocatalytic activity in addition to their low fabrication cost.^39^, ^40^, ^41^

## 2 Results and Discussion

**Figure 1:**
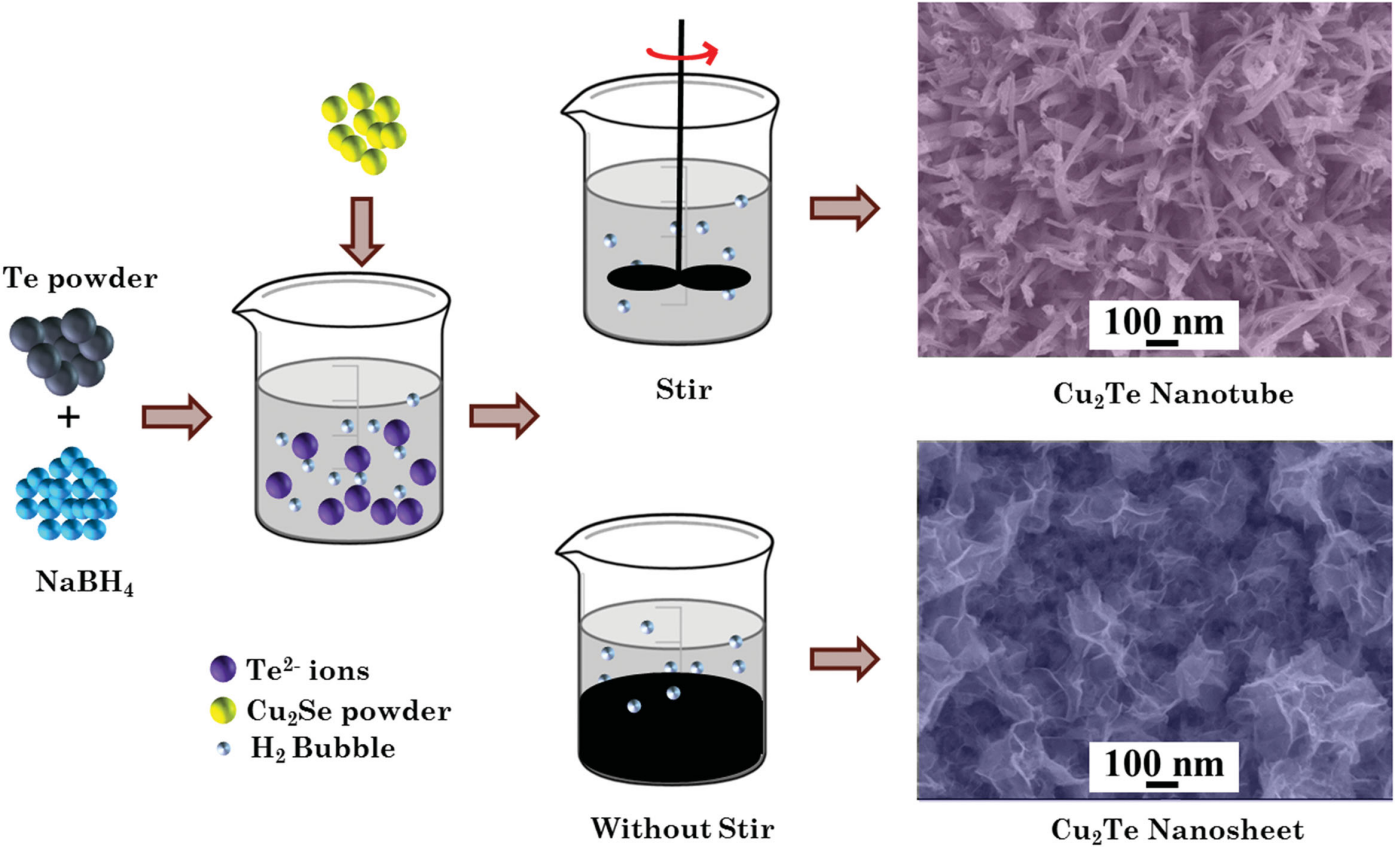
Schematic illustration of the synthesis of Cu_2_Te nanosheets and nanotubes from Cu_2_Se nanoparticles.

**Figure 2:**
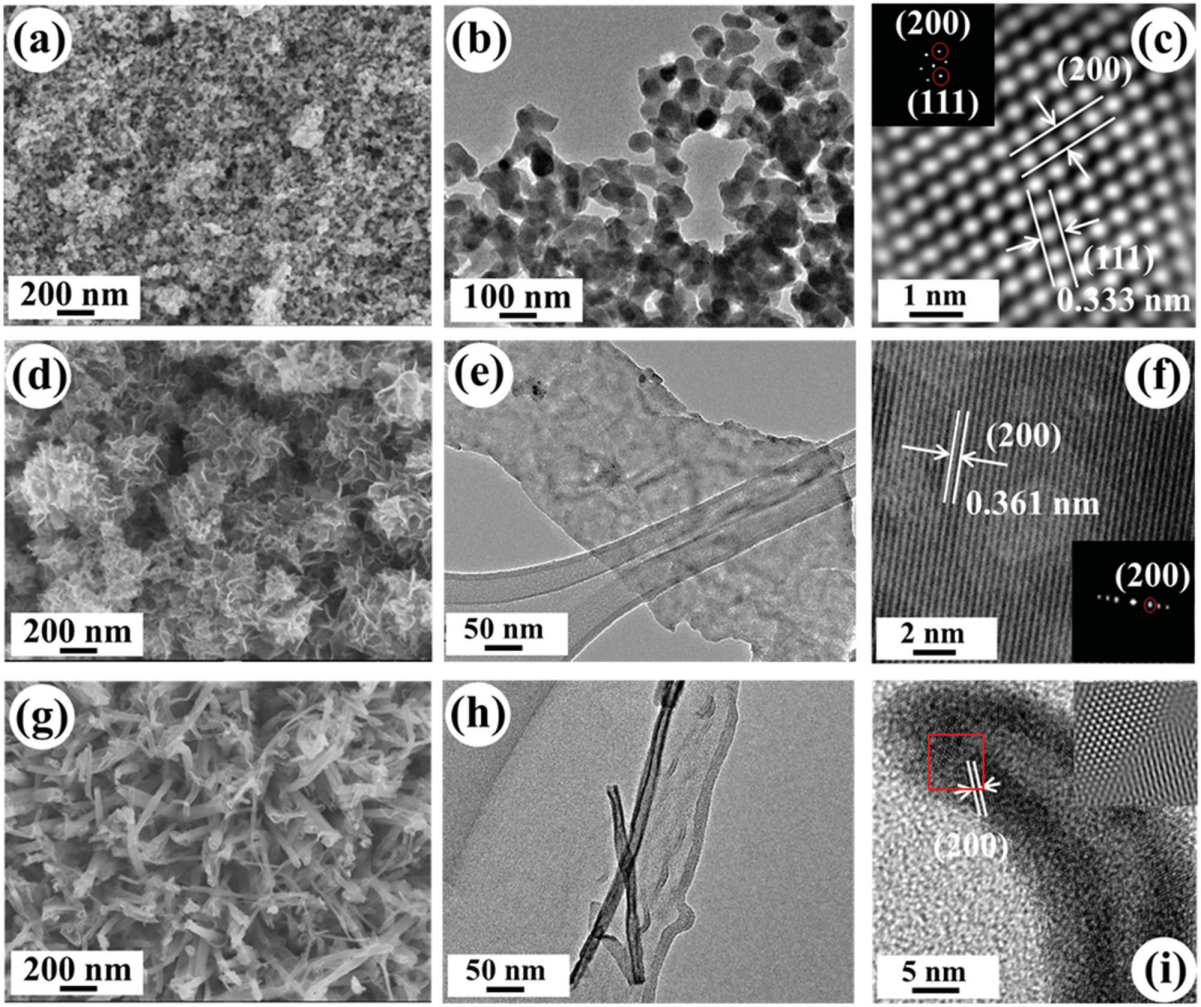
a) SEM, b) TEM, and c) HRTEM images of Cu_2_Se nanoparticle precursor; d) SEM, f) TEM, and c) HRTEM images of the as‐prepared Cu_2_Te nanosheets; g) SEM, h) TEM, and i) HRTEM images of Cu_2_Te nanotubes. The insets to (c) and (f) are the corresponding FFT patterns; the inset in (i) is the IFFT image of the part marked by red rectangle.

The Cu_2_Se precursor and final precipitates prepared from the schedule in **Figure** **1** were collected and characterized, respectively. Their morphologies were elucidated in **Figure** **2**; and the compositions were verified by the XRD patterns shown in Figure S1 (Supporting Information), which can be unambiguously indexed to Cu_2_Se (JCPDS 06‐0680) and Cu_2_Te (JCPDS 10‐0421), respectively. As shown in Figure 2a–c, Cu_2_Se precursor is made up of uniform nanoparticles ranging from 10 to 20 nm in size, whose lattice and FFT pattern can be well indexed to be face‐centered cubic (fcc) Cu_2_Se. After being added into the Na_2_Te solution and kept stationary for 30 min, the Cu_2_Se nanoparticles are transformed into flower like nanosheets (Figure 2d,e). The HRTEM and FFT images show a clear lattice of (220) planes of Cu_2_Te (Figure 2f), while rolled tubular nanostructures are observed if the turbid mixture of Cu_2_Se and Te precursor is well stirred, as shown in Figure 2g–i. This result demonstrates that Cu_2_Te nanosheets and nanotubes can be effectively synthesized via a simple and fast anion exchange from Cu_2_Se nanoparticles in aqueous solution at room temperature without any surfactants. The whole reaction procedure can be expressed by Equations (1) and (2)

(1) $$2\mathrm{Te}+4{\mathrm{NaBH}}_{4}+7H_{2}O\to 2{\mathrm{Na}}_{2}\mathrm{Te}+14H_{2}↑+\;\;H_{2}B_{4}O_{7}$$

(2) $${\mathrm{Cu}}_{2}\mathrm{Se}+{\mathrm{Te}}^{2-}\to {\mathrm{Cu}}_{2}\mathrm{Te}+{\mathrm{Se}}^{2-}$$

In contrast to the similar anion exchange reactions of its analogues, such as from Cu_2_O to Cu_2_S,^42^, ^43^, ^44^, ^45^, ^46^ however, the transfer reaction between Cu_2_Se and Cu_2_Te exhibits distinct features, such as the different ambient reaction conditions and the ultrafast reaction speed, as well as the enormous differences in the morphology of the Cu_2_Se nanoparticles and the Cu_2_Te nanosheets [i.e., from the small zero‐dimensional (0D) particle structure to the large 2D sheet structure], which cannot be simply explained by the Kirkendall effect.

To better understand the anomalous features of this exchange process, both thermodynamic and kinetic reasons were considered. First of all, as the exchange was conducted in aqueous solution, the Gibbs free energy (ΔG) for insoluble materials can be expressed by Equation (3), in which *R* is the gas constant, *T* is the temperature, and *K*_sp_ is the solubility of product. *J*_sp_ is expressed by Equation (4), where C(Cu^+^) and C(Se^2−^) represent the concentrations of Cu^+^ and Se^2−^, respectively.

(3) $$\Delta G\;=\;RT\;\;\;\times \;\;\;ln\frac{J_{\mathrm{sp}}}{K_{\mathrm{sp}}}$$

(4) $$J_{\mathrm{SP}}=C^{2}\;\left({\mathrm{Cu}}^{+}\right)\;\;\times \;\;\;C\left({\mathrm{Se}}^{2-}\right)$$

The driving force of Equation (3) originates from the difference between the solubility (*K*_sp_) of Cu_2_Se and Cu_2_Te in aqueous solution.^20^, ^21^, ^22^, ^23^, ^24^ Although the solubility of Cu_2_Te is unavailable, according to the literature,^47^ the solubility of metal chalcogenides always decrease with increasing ionic radius of the chalcogen element, i.e., *K*_sp_(Cu_2_O) >* K*_sp_(Cu_2_S) > *K*_sp_(Cu_2_Se) > *K*_sp_(Cu_2_Te) (Table S1, Supporting Information); and we can infer that the driving force for the conversion from Cu_2_E (E = O, S, Se) to Cu_2_Te has the same tendency, e.g., Cu_2_O > Cu_2_S > Cu_2_Se. The inference that transfer reactions from Cu_2_O/Cu_2_S/Cu_2_Se to Cu_2_Te are all energy favorable has been confirmed by the results shown in Figure S2 (Supporting Information). In addition, because the structure of Cu_2_Se (fcc) is totally different from the hexagonal structure of Cu_2_Te, the derived energy differences, as well as the surface energy changes, play a pivotal role in the activation barrier of the transfer reaction (Table S1, Supporting Information).

**Figure 3:**
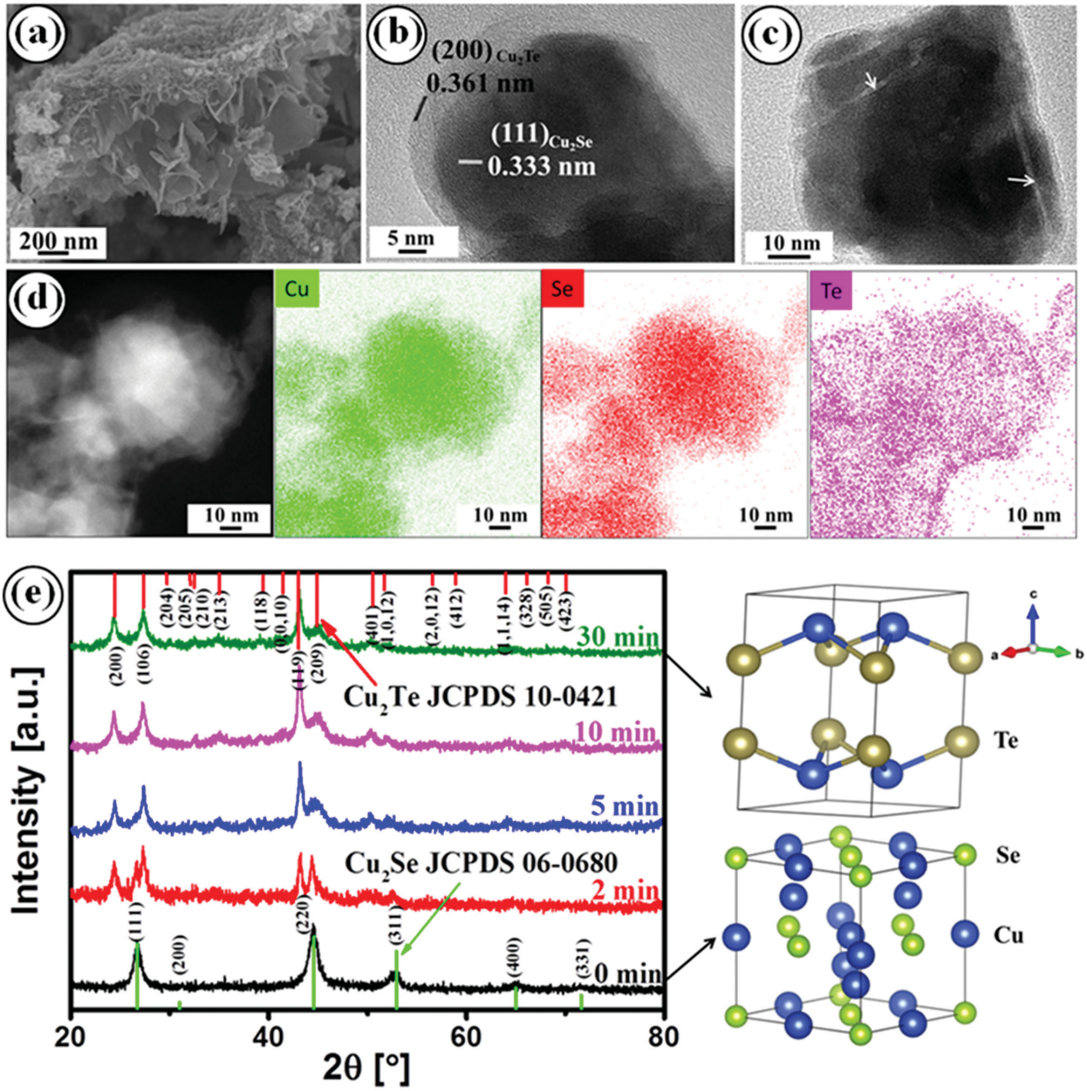
a–c) SEM and TEM images of the precipitate after reaction of 2 min; d) EDS mapping of the precipitate after reaction of 2 min; e) XRD patterns of the precipitates obtained from different reaction times, right side shows crystal structures of hexagonal Cu_2_Te (top) and fcc Cu_2_Se (bottom), respectively.

To investigate the transfer procedure from Cu_2_Se nanoparticles to Cu_2_Te nanosheets, a series of control experiments were conducted, and the detailed parameters are listed in Table S2 (Supporting Information). In the first group of experiments (Group E1), the reaction was slowed down by an ice bath and by diluting the Te precursor to 0.01 m. After the addition of Cu_2_Se precursor for 2, 5, 10, and 30 min, respectively, some of the turbid suspension was taken out, and water was immediately remove by a suction filter to stop the reaction. The precipitate was then characterized after being washed several times with absolute ethanol. **Figure** **3**a shows a typical SEM image of the sample taken out after 2 min: both nanoparticles and nanosheets can be identified. Figure 3b,c shows the typical TEM images of this sample. A core–shell structure is clearly observed in Figure 3b, the planar spacing of the inner darker part (core) is 0.333 nm, which can be indexed to the (111) planes of fcc Cu_2_Se. The outer layered fringe shows a planar spacing of 0.361 nm, which coincides with the (200) planes of hexagonal Cu_2_Te. Thus, Cu_2_Se nanoparticles are wrapped by thin Cu_2_Te layers to form core–shell Cu_2_Se@Cu_2_Te structure after reaction of 2 min. The composition of a similar structure was further identified by EDS mapping conducted on a JOEL ARM‐200F (Figure 3d), and the results show that the Se element is mainly distributed in the inner bright part, while the Te element covers the whole area, supporting that Cu_2_Se was wrapped by Cu_2_Te. In addition, obvious cracks (marked by white arrows) are observed in some particles as displayed in Figure 3c. The X‐ray diffraction patterns of the samples taken out after different reaction times are presented in Figure 3e. In accordance with the SEM and TEM results, after 2 min reaction, the diffraction peaks of both hexagonal Cu_2_Te (JCPDS 10‐0421) and fcc Cu_2_Se (JCPDS 06‐0680) are detected; while after 5 min, the transfer reaction from Cu_2_Se to Cu_2_Te is almost completed, as indicated by vanishing of the Cu_2_Se peaks (JCPDS 06‐0680). The XPS results presented in Figure S3 (Supporting Information) are consistent with the XRD results. From comparison of the peaks of our samples with the literature,^12^, ^48^ it is easy to identify that before the reaction started (0 min), the composition of the precursor was pure Cu_2_Se.^48^ After 2 min reaction, the Se^2−^ peaks are still observed with appearance of Te peaks. The peaks at 571.9 and 582.5 eV can be assigned to the peaks of Te^2−^; while the other two peaks located at 575.4 and 585.8 eV are the peaks from oxidation of the sample.^12^ After reacting for 30 min, the peaks of Se totally disappeared, and the composition of the final product becomes pure Cu_2_Te. As mentioned previously, the crystal structure of product Cu_2_Te (JCPDS 10‐0421) is different to that of Cu_2_Se precursor (JCPDS 06‐0680). Cu_2_Te shows an obvious layered structure in Figure 3e, and the calculated volume expansion (ΔV/V_Cu2Se_) for Cu_2_Se converted into Cu_2_Te is as high as 91.53% (Table S1, Supporting Information) for each Cu_2_Se unit cell.

**Figure 4:**
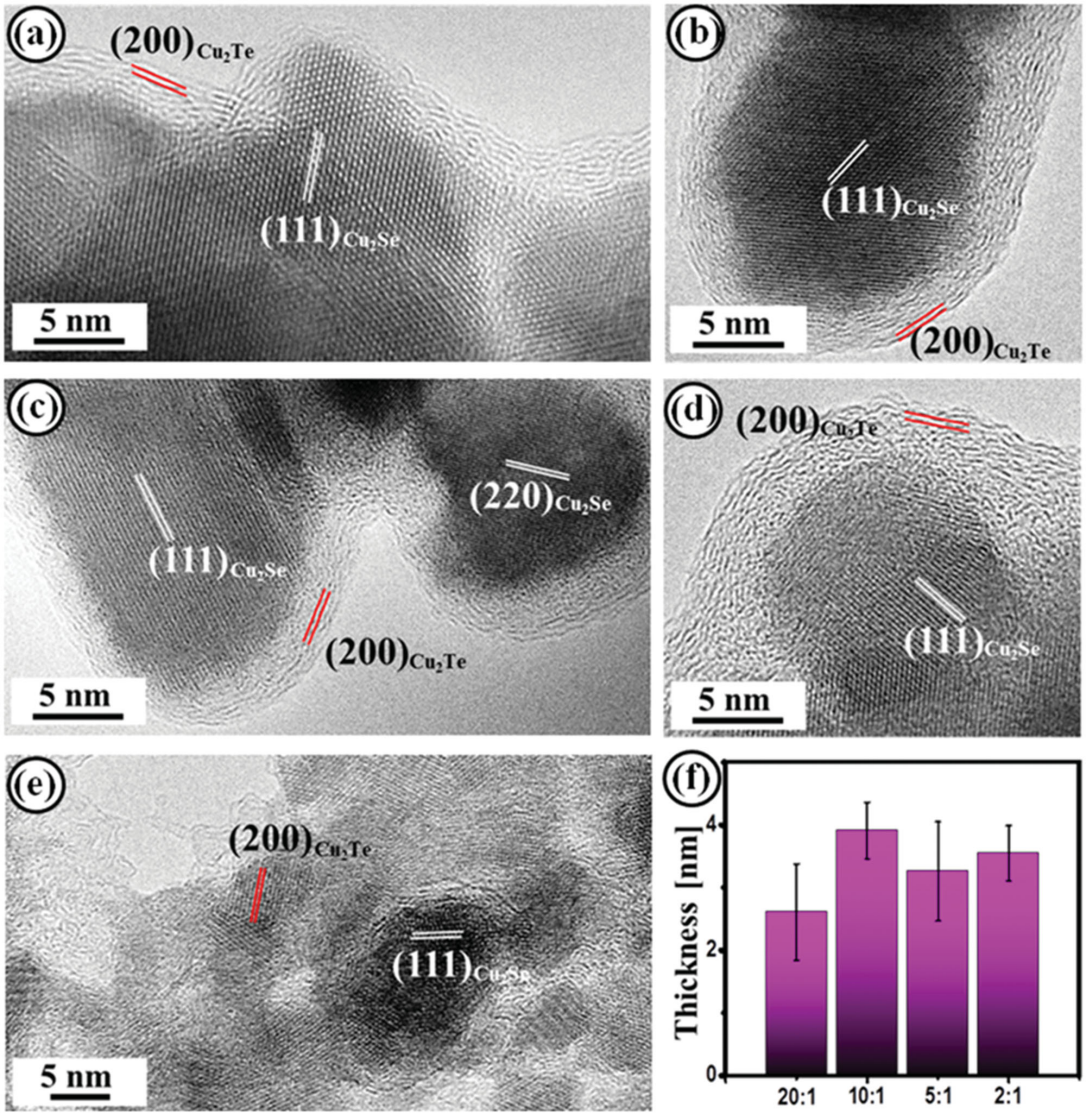
HRTEM images of the samples obtained from different mole ratios of Cu_2_Se:Te: a) 20:1; b) 10:1; c) 5:1; and d) 2:1. e) Hybrid structure consisting of sheets and nanoparticles in the sample obtained from a ratio of 10:1. f) Statistical thickness of Cu_2_Te layers in (a–d).

To further clarify the evolution mechanism, another group of control experiments was conducted (Group E2 in Table S2, Supporting Information). The molar ratio between the Cu_2_Se precursor and Te was varied from 20:1 to 2:1, and the thickness of the Cu_2_Te layers wrapped around Cu_2_Se nanoparticles was investigated by TEM. The results are presented in **Figure** **4**a–d. It is obvious that there are both uniform and continuous layers wrapping the particles in these sample, and the lattice spacing of the inner and outer parts can be indexed to (111) of Cu_2_Se and (200) of Cu_2_Te, respectively.

Meanwhile, a hybrid structure consisting of nanosheets and nanoparticles can be easily detected in the sample obtained from a ratio of 10:1, as shown in Figure 4e. The statistics on the thickness of outer layers are presented in Figure 4f, which does not monotonically increase with decreasing Cu_2_Se to Te ratio. The thickness of Cu_2_Te layer first increases from 2.61 ± 0.77 to 3.91 ± 0.45 nm, then decreases to 3.26 ± 0.79 nm, and finally increases again to 3.55 ± 0.44 nm as the molar ratio of Cu_2_Se to Te decreases from 20:1 to 2:1. Combining the cracks shown in Figure 3c and the hybrid structure presented in Figure 4e, we can deduce that a peeling of the Cu_2_Te layers occurred when their thickness beyond 4 nm.

**Figure 5:**
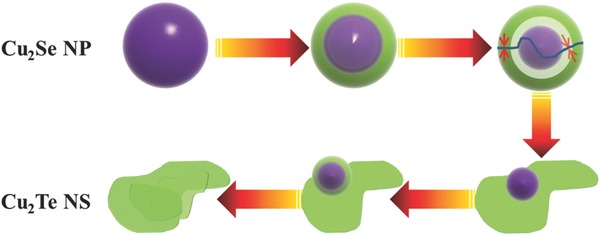
Schematic illustration of the transfer reaction from Cu_2_Se nanoparticles to Cu_2_Te nanosheets.

Based on the above results, the transfer procedure from Cu_2_Se nanoparticles to Cu_2_Te nanosheets was schematically illustrated in **Figure** **5**. When Cu_2_Se nanoparticles are added into the Te^2−^ solution, the particle surface Cu^+^ ions reacts with Te^2−^ ions immediately to form a layer of hexagonal Cu_2_Te, due to the lower solubility of Cu_2_Te than Cu_2_Se. With the formation of Cu_2_Te layer, the Cu^+^ ions diffusing outward and the Te^2−^ ions diffusing inward react at the interface between core and shell to form new Cu_2_Te and increase the thickness of Cu_2_Te layer. Benefiting from the large volume expansion [Table S1 (Supporting Information), ΔV/V_Cu2Se_ = 91.53%] between fcc Cu_2_Se and hexagonal Cu_2_Te, tension and defects accumulate with the growth of the Cu_2_Te layer, finally leading to peeling of Cu_2_Te from Cu_2_Se.^47^ As long as the mass transport between Cu_2_Se and Cu_2_Te exists, the growth and peeling of Cu_2_Te would continue until Cu_2_Se is completely converted. The peeled Cu_2_Te layers can grow into large nanosheets because of unique layered structure of Cu_2_Te (Figure 3e). As shown in Figure 2g–i, the obtained Cu_2_Te nanosheets could be further rolled into Cu_2_Te nanotubes through the effects of continuous stirring and of tension (including both surface tension of the nanosheets and tension in the crystals). The conversion process can be summarized into two steps. The first step is a diffusion process related Kirkendall effect to form Cu_2_Te shell, which is similar to the transfer reactions between Cu_2_E (E = O, S, Se) compounds;^42^, ^43^, ^44^, ^45^, ^46^ the second step is the peeling and continued growth of the Cu_2_Te layer into nanosheets as proved by Figures 3c and 4.

Similarly, Cu_2_O and Cu_2_S nanoparticles can be converted into Cu_2_Te nanosheets. The volume changes between Cu_2_O, Cu_2_S, and Cu_2_Te reach 479% and 229%, respectively (Table S1, Supporting Information). Due to the pronounced differences in their *K*_sp_ (Table S1, Supporting Information), the conversion of Cu_2_O and Cu_2_S nanoparticles into Cu_2_Te nanosheets is easier than the conversion of Cu_2_Se nanoparticles. Compared with conversion of Cu_2_E (E = O, S, Se) into Cu_2_Te, the volume expansions between them (i.e., Cu_2_O and Cu_2_S, Cu_2_O and Cu_2_Se, and Cu_2_S and Cu_2_Se) are much less and reach 17.5%, 42.8%, and 6.6%, respectively. Therefore, the anion exchange between Cu_2_E (E = O, S, Se) nanoparticles can preserve the original morphology and can be simply explained by the Kirkendall effect.^42^, ^43^, ^44^, ^45^, ^46^

**Figure 6:**
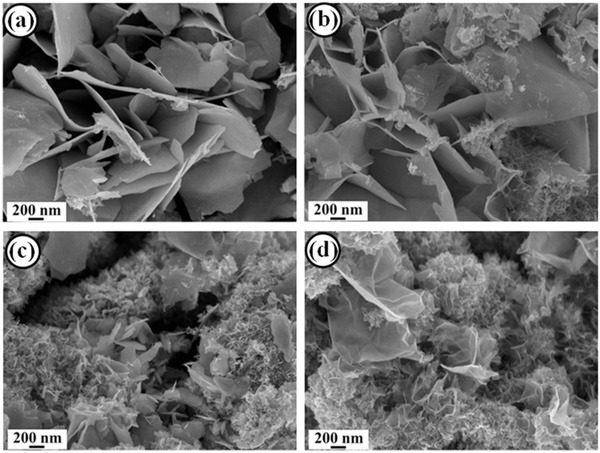
SEM images of Cu_2_Te nanosheets prepared from different concentrations of Na_2_Te: a) 0.1 m; b) 0.05 m; c) 0.02 m; d) 0.01 m.

Another group of control experiments (Group E3 in Table S2, Supporting Information,) were conducted to verify the effects of the Te^2−^ concentration on the thickness of the Cu_2_Te nanosheets. As illustrated by the SEM images of samples shown in **Figure** **6**a–d, the concentration of Te^2−^ had a significant impact on the morphology of the final Cu_2_Te. When the concentration of Te^2−^ decreased from 0.1 to 0.05, 0.02, and 0.01 m, the thickness of the Cu_2_Te nanosheets obviously decreased, and they became more and more flexible. The Cu_2_Te nanosheets also became smaller and more inhomogeneous. This phenomenon could be well explained by the formation mechanism of the nanosheets (Figure 5). When the concentration of Te^2−^ is higher (e.g., 0.1 m), a thicker initial Cu_2_Te layer formed in a very short time, which could yield a homogeneous large and thick nanosheets (Figure 6a). On the contrary, if the concentration of Te^2−^ is lower, a thinner Cu_2_Te layer is formed at the initial stage; while multipeeling from nanoparticles leads to smaller and inhomogeneous nanosheets (Figure 6b–d).

As the transfer reaction [Equation (2)] is a solid‐solution reaction, the size of Cu_2_Se nanoparticle precursor also plays an important role in the morphologies of Cu_2_Te nanosheets. To investigate this effect, we first synthesized four batches of Cu_2_Se nanoparticles [Group E4 in Table S3, Supporting Information] with different sizes according to the literature.^48^ The XRD patterns, SEM images, TEM images, and size distributions of the four Cu_2_Se precursors are respectively presented from Figures S4–S7 in the Supporting Information. The morphologies of thus‐formed Cu_2_Te nanosheets are presented in Figure S8 (Supporting Information), in which it is hard to see any difference. To reveal the impact, the thickness of Cu_2_Te nanosheets fabricated from different Cu_2_Se precursors was determined by AFM (Figure S9a–d, Supporting Information). With increasing size of the Cu_2_Se precursor, the average thickness measured from tens pieces of Cu_2_Te nanosheets are 4.2 ± 0.7, 3.7 ± 0.5, 4.3 ± 0.9, and 3.2 ± 0.4 nm for E4‐1 to E4‐4, respectively. The thickness of Cu_2_Te nanosheets varied slightly. The surface area of Cu_2_Se nanoparticles and Cu_2_Te nanosheets were analyzed by BET analysis, and the results are plotted in Figure S10 (Supporting Information). With increasing Cu_2_Se nanoparticle size from 11.9 to 25.5 nm, its specific surface area decreased from 38.4 to 21.2 m^2^ g^−1^. After being transformed into Cu_2_Te nanosheets, the specific area increased to a different extent. For example, the maximum ratio appeared in Cu_2_Se nanoparticles with a size of 25.5 nm (E4‐4), and the specific surface area increased from 21.2 to 32.8 m^2^ g^−1^, while in the case of 22.5 nm Cu_2_Se (E4‐3), the specific surface area increased slightly from 22.4 to 23.5 m^2^ g^−1^.

**Figure 7:**
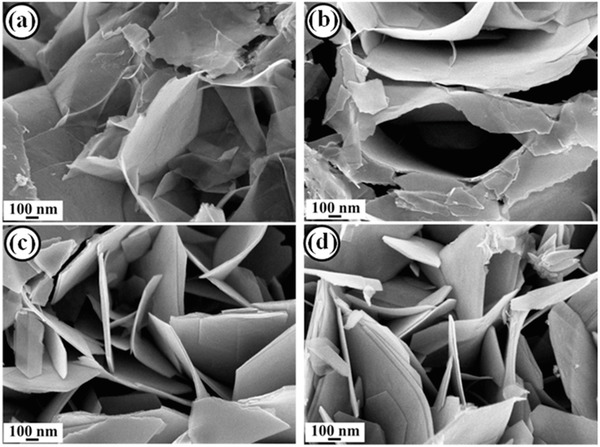
Cu_2_Te nanosheets prepared with different mole ratios of NaBH_4_ to Te. a) 9:1; b) 18:1; c) 36:1; d) 80:1.

Another factor influencing the morphology of the final product is the amount of NaBH_4_. When the mole ratio between NaBH_4_ and Te is raised from 9:1 to 18:1, 36:1, and 80:1 [Group E5 in Table S2, Supporting Information], we can conclude from **Figure** **7** that the thickness of the Cu_2_Te nanosheets increases. This result is related to the property of Se^2−^, which is very easy to hydrolyze in aqueous solution [Equation (5)],^49^, ^50^, ^51^ while extra NaBH_4_ will lead to an increased pH value due to the formation of NaBO_2_ [Equation (6)–(7)].^48^ Although Te^2−^ could also hydrolyze into HTe^−^ as shown in Equation (8), the hydrolysis is much weaker than Se^2−^ as pK_b_(HSe^−^) = 10.2 < pK_b_(HTe^−^) = 11.4. As a result, the increase of solution pH would suppress the conversion of Cu_2_Se according to Equation (9), leading to slow growth and less defects of Cu_2_Te nanosheets.

(5) $${\mathrm{Se}}^{2-}+H_{2}O\to {\mathrm{HSe}}^{-}+{\mathrm{OH}}^{-}$$

(6) $${\mathrm{NaBH}}_{4}+2H_{2}O\to {\mathrm{NaBO}}_{2}+4H_{2}↑$$

(7) $${\mathrm{BO}}_{2}^{-}+H_{2}O\to {\mathrm{HBO}}_{2}+{\mathrm{OH}}^{-}$$

(8) $${\mathrm{Te}}^{2-}+H_{2}O\to {\mathrm{HTe}}^{-}+{\mathrm{OH}}^{-}$$

(9) $${\mathrm{Cu}}_{2}\mathrm{Se}+H_{2}O+{\mathrm{Te}}^{2-}\to {\mathrm{Cu}}_{2}\mathrm{Te}+{\mathrm{OH}}^{-}+{\mathrm{HSe}}^{-}$$

As seen from Figure 2h, the Cu_2_Te nanotubes are generated by rolling of the formed Cu_2_Te nanosheets with assistance of stirring, the effect of stirring speed on the properties of nanotubes was investigated, and the detailed experimental conditions are presented in Table S2 (Supporting Information) (Group E6). As shown in Figure S11 (Supporting Information), the length of the rolled nanotubes gradually decreased with the stirring speed increasing from 100 to 1000 rpm.

**Figure 8:**
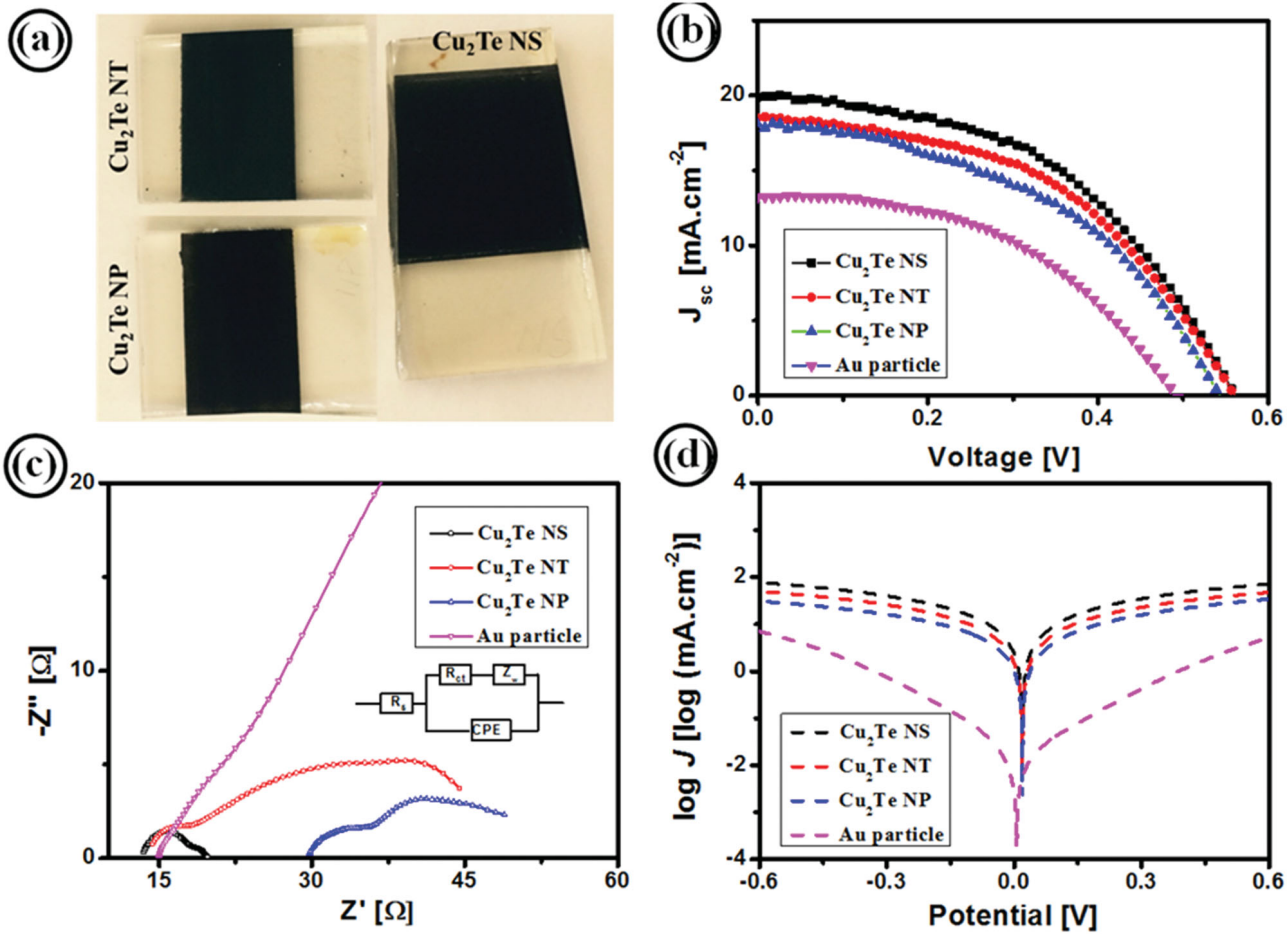
a) Photograph of the assembled Cu_2_Te CEs; b) *J–V* curves for the different CEs; c) electrochemical impedance spectra for the CEs, with the inset showing the equivalent circuit; d) Tafel‐polarization curves for the CEs.

To test the potential application of obtained Cu_2_Te nanosheets (Table S2, E1‐4, Supporting Information) and nanotubes (Table S2, E6‐1, Supporting Information) as counter electrodes (**Figure** **8**a) in solar cells, CdS/CdSe cosensitized solar cells were assembled. Cu_2_Te nanoparticle based counter electrodes were also prepared to investigate the effects of morphology on performance. The preparation and characterizations of Cu_2_Te nanoparticles are detailed in Part 3 and Figure S12a–d in the Supporting Information, respectively. As a reference, an Au film counter electrode was also fabricated. The photocurrent density–voltage (*J*–*V*) curves of the 4 CEs (Cu_2_Te‐NS CE, Cu_2_Te‐NT CE, Cu_2_Te‐NP CE, and Au CE) under standard simulated AM 1.5 illumination are shown in Figure 8b. The corresponding photovoltaic performance parameters are summarized in **Table** **1**. The Cu_2_Te‐NS CE shows the highest short‐circuit current of 19.87 mA cm^−2^, with open‐circuit voltage (*V*_oc_) of 0.57 V, and fill factor (FF) of 0.472, yielding the highest power conversion efficiency (PCE, *η*) of 5.35%. The Cu_2_Te‐NT CE exhibits a conversion efficiency of 4.75%, a lower *J*_sc_ (18.49 mA cm^−2^), and *V*_oc_ (0.56 V) compared with Cu_2_Te‐NS CE, despite its slightly higher FF of 0.476. The performance of Cu_2_Te‐NP CE is inferior to that of the Cu_2_Te‐NT sample due to its smaller *J*_sc_ (18.13 mA cm^−2^), *V*_oc_ (0.546 V), and fill factor (0.452). The Au CE presents the lowest *J*_sc_ (13.20 mA cm^−2^) and *V*_oc_ (0.486 V), but the highest fill factor (0.492) among the 4 CEs, respectively. The conversion efficiency of solar cells made with Au CE only reaches 3.16%, which is obviously lower than that of solar cells with Cu_2_Te CEs. It is safe for us to conclude that our low‐cost Cu_2_Te CEs yield better performance than noble Au CEs. It is also clear that the morphology of Cu_2_Te influenced the performance of solar cells.

**Table 1:** Photovoltaic performancea) of QDSSCs with different CEs and EIS parametersb) in cells assembled with the same CdS/CdSe QD working electrodes

Sample	*J*_sc_ [mA cm^−2^]	*V*_oc_ [Voltage]	Fill factor	*η* [%]	*R*_s_ [Ω]	*R*_ct_ [Ω]	*Z*_w_ [Ω]
Cu_2_Te NS	19.87	0.57	0.472	5.35	13.21	5.08	3.9
Cu_2_Te NT	18.49	0.56	0.476	4.75	13.73	5.93	33.2
Cu_2_Te NP	18.13	0.546	0.452	4.47	29.24	8.41	23.0
Au	13.20	0.492	0.486	3.16	14.97	46.25	73.4

^a)^ *J*_sc_: short‐circuit current; *V*_oc_: open‐circuit voltage; *η*: energy conversion efficiency;

^b)^ *R*_s_: series resistance; *R*_ct_: charge transfer resistance at the CE/electrolyte interface; *Z*_w_: Warburg impedance.

To further clarify the electrochemical characteristics of various Cu_2_Te‐based CEs in catalyzing the redox couple of the electrolyte ($S^{2-}/S_{n}^{2-}$), electrochemical impedance spectroscopy (EIS, Figure 8c) and Tafel‐polarization measurements (Figure 8d) were carried out. The Nyquist plots for the dummy cells with different CEs plotted in Figure 8c with the equivalent circuit are used to interpret the spectra. The impedance spectra of the CEs can be modeled by several circuit elements, such as for series resistance (*R*_s_), charge transfer resistance (*R*_ct_), and Warburg diffusion impedance (*Z*_W_). The value of *R*_s_ can be evaluated from the high‐frequency intercept on the real axis, which comprises the bulk resistance of the CE material, the resistance of the substrate, and the contact resistance. The *R*_ct_ can be obtained by fitting the arc in the middle‐frequency region, which corresponds to the charge‐transfer process at the CE/electrolyte interface. This value is a direct indicator of catalytic activity. The arc in the low‐frequency range indicates *Z*_W_, which arises from diffusion of the electrolyte.^30^, ^32^, ^52^ All these parameters were determined by fitting the impedance spectra using the Z‐view software and are summarized in Table 1.

**Figure 9:**
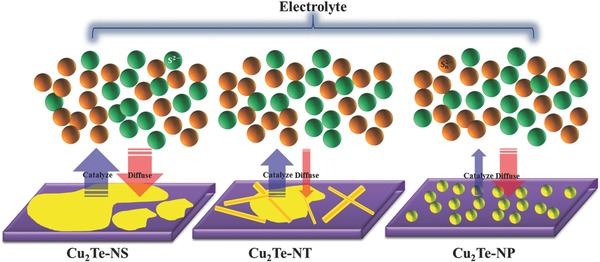
Schematic illustration of different Cu_2_Te CEs.

As enumerated in Table 1, the *R*_ct_ values of Cu_2_Te‐NS CE (5.08 Ω), Cu_2_Te‐NT CE (5.93 Ω), and Cu_2_Te‐NP CE (8.41 Ω) are far below that of the Au CE (46.25 Ω), which indicates their excellent catalytic activity toward the $S^{2-}/S_{n}^{2-}\;$ redox couple. This is consistent with the increased *J_sc_* and *V_oc_* displayed in Table 1. The superior catalytic property of Cu_2_Te‐NS CE is mostly due to the higher surface area which enables much more active sites for the catalytic redox reaction. This was verified by the BET results shown in Figure S13 (Supporting Information) and **Figure** **9**. The Cu_2_Te‐NS sample possesses the highest specific surface area (36.6 m^2^ g^−1^), while the specific surface area of the Cu_2_Te‐NP sample is the lowest (22.5 m^2^ g^−1^). In addition, the *R*_s_ of Cu_2_Te‐NS and Cu_2_Te‐NT CE, which reached 13.21 and 13.73 Ω, respectively, is obviously lower than for Cu_2_Te‐NP CE (29.24 Ω) and close to Au CE.

The difference in the performance of nanostructured Cu_2_Te CEs could be attributed to their different morphology. In the Cu_2_Te nanoparticle (0‐dimentional) film, charge carries were transported through a zigzag way and readily trapped by surface defects, leading to lower conductivity. The series resistance was significantly reduced in both Cu_2_Te‐NT and Cu_2_Te‐NS CEs, which is owing to the better carrier pathway provided by 1D nanotubes and 2D nanosheets shown in Figure 9. The lower series resistance would lead to higher FF and *V*_oc_. The diffusion resistances for the electrolyte have been evaluated from the values of *Z*_W_ and are shown in Table 1. The *Z*_W_ of Cu_2_Te‐NS CE (3.9 Ω) is significantly lower than for the other three CEs, indicating much easier and faster diffusion of the electrolyte. The *Z*_W_ value of Cu_2_Te‐NT CE reaches 33.2 Ω, which is the highest among the three Cu_2_Te CEs. This may be due to difficult diffusion of electrolyte into the nanotubes. The high value of *Z*_W_ decreased the overall energy conversion efficiency (*η* = 4.75%) of solar cells made with Cu_2_Te‐NT CE. Compared with the other two Cu_2_Te CEs, Cu_2_Te‐NP CE shows the highest *R*_s_ and *R*_ct_, and a moderate *Z*_W_ (23.0 Ω). It thus possesses the lowest fill factor (0.452) and conversion efficiency of 4.47%. The highest *Z*_W_ value (73.4 Ω) is achieved with the Au CE.

The Tafel‐polarization curves of the cells used for EIS measurements are plotted in Figure 8d. The curves represent the logarithm of the current density (logJ) as a function of the potential (V) for the $S^{2-}/S_{n}^{2-}$ redox couple of the electrolyte. The slopes for the electrodes indicate the exchange current density (*J*_0_), which is related to *R*_ct_ in terms of Equation (10). *R*, *T*, *F*, and *n* respectively represent the gas constant, temperature, Faraday constant, and number of electrons involved in the redox reaction of the electrolyte; while *R*_ct_ is the charge transfer resistance. As shown in Figure 8d, the slopes of the Tafel curves for the four CEs are in the order of Cu_2_Te‐NS > Cu_2_Te‐NT > Cu_2_Te‐NP > Au, which coincides with the order of the *R*_ct_ values listed in Table 1

(10) $$J_{0}=\;RT/\left(nFR_{\mathrm{ct}}\right)$$

## 3 Conclusion

We have developed a fast, aqueous, cost‐effective, and green strategy to prepare Cu_2_Te nanotubes and nanosheets from Cu_2_E (E = O, S, Se) nanoparticles at room temperature, which can be ascribed to an exchange‐peeling mechanism and the unique crystal structure of Cu_2_Te. The morphologies of the Cu_2_Te nanosheets and nanotubes could be easily controlled by adjusting the precursor concentrations, particle size of the Cu_2_Se precursor, and the amount of NaBH_4_, as well as the stirring speed. Thus‐formed Cu_2_Te nanostructures were used as counter electrodes in CdS/CdSe cosensitized solar cells and show excellent catalytic activity toward the electrolyte compared with expensive Au counter electrodes. The performance of Cu_2_Te strongly depends on its morphology, in which Cu_2_Te nanosheets showed the maximum conversion efficiency of 5.35% and Cu_2_Te nanoparticles showed a value of 4.47%. Considering their low‐cost and high‐catalytic activity, these Cu_2_Te nanostructures have great potential in energy conversion.

## 4 Experimental Section

*Synthesis of Cu_2_Te Nanotubes and Nanosheets*: As illustrated in Figure 1, a typical synthesis of Cu_2_Te nanotubes is as follows: 127 mg of tellurium powder (1 mmol, 100 mesh, ≥99.5%) and 3 g of NaBH_4_ (caplets, ≥98%) were added into 100 mL of Milli‐Q water, with continuous stirring under the protection of inert gas. Several minutes later, a light purple (or colorless) Te precursor solution formed. The Cu_2_Se nanoparticle precursor was prepared according to a previous report.^48^ First, 1 mmol of selenium powder (100 mesh, ≥99.5%) was reduced with 3 mmol of NaBH_4_ in distilled water for 30 min, then precipitated with a 10 mL, 0.2 m CuCl_2_·2H_2_O (≥99%) solution. The as‐obtained Cu_2_Se nanoparticles were purified, dried, and ultrasonically dispersed into 40 mL H_2_O. Then, the dispersed turbid suspension was mixed with the Te precursor at room temperature, followed by continuous stirring for 30 min under protection of inert gas, and Cu_2_Te nanotube structures were obtained. To form the Cu_2_Te nanosheets, after the solution was mixed with the turbid suspension, the stirring was immediately stopped, and the suspension was simply held for 30 min.

Cu_2_Te nanotubes and nanosheets can be also synthesized by the above procedure from Cu_2_O nanoparticles and Cu_2_S nanoparticles. The synthesis of Cu_2_O and Cu_2_S nanoparticles is similar to Cu_2_Se.^48^ Cu_2_S was obtained by first mixing 1 mmol of Na_2_S·9H_2_O (≥99.5%) with 1 mmol of NaBH_4_ in distilled water for 30 min, then precipitating it with 10 mL, 0.2 m CuCl_2_·2H_2_O (≥99%) solution. Cu_2_O was simply prepared by adding a 10 mL, freshly prepared 0.1 m solution of NaBH_4_ to 10 mL, 0.2 m CuCl_2_·2H_2_O solution.

*Characterization*: X‐ray diffraction (XRD) studies were carried out at room temperature with an X‐ray diffractometer (GBC‐MMA) using Cu‐K_α_ radiation (*λ* = 0.154 nm). Scanning electron microscopy (SEM) was conducted on a JEOL JSM‐7500FA microscope. Transmission electron microscopy (TEM) was performed using a JEOL JEM‐2011 microscope with an accelerating voltage of 200 kV. Scanning transmission electron microscopy‐energy dispersive X‐ray spectroscopy (STEM‐EDS) spectra were collected using a JEOL ARM‐200F operating at 200 kV with an EDAX solid‐state X‐ray detector. Samples were prepared by drop‐casting ethanol‐dispersed nanostructures onto TEM copper grids. Lattice fringes were measured from the fast Fourier transforms (FFTs) of high resolution TEM (HRTEM) images using the Gatan software. Atomic force microscope (AFM) images were collected using an Asylum AFM equipped with NCHR tapping mode. AFM tips were bought from the “Nanoworld” Company. Nitrogen adsorption‐desorption measurements were conducted at 77 K with a Quantachrome Autosorb iQ machine (USA). Before measurements, the samples were degassed under vacuum at 120 °C for 6 h. The Brunauer–Emmett–Teller (BET) specific surface area was calculated from the adsorption data in the relative pressure (P/P_0_) range from 0.05 to 0.35. X‐ray photoelectron spectroscopy (XPS) was conducted using a SPECS PHOIBOS 100 Analyzer installed in a high‐vacuum chamber with the base pressure below 10^–8^ mbar. X‐ray excitation was provided by Al K_α_ radiation with the photon energy hν = 1486.6 eV at the high voltage of 12 kV and power of 120 W. All the spectra were calibrated with respect to C 1s at 284.6 eV.

*Fabrication and Testing of CdS/CdSe Cosensitized Solar Cell*: Preparation of photoelectrodes: CdS/CdSe quantum dots (QDs) cosensitized TiO_2_ working electrodes were prepared by a previously developed technique.^29^, ^30^, ^41^ The Cu_2_Te counter electrodes (CEs) were fabricated by depositing various pastes on fluorine‐doped tin oxide (FTO) substrates using the doctor blade technique.^41^, ^52^ Then, the newly formed films were annealed at 350 °C for 30 min in Argon atmosphere. For comparison, Au electrodes were prepared by sputtering a thickness of ≈50 nm film on the substrate (obtained from the sputtering calibration curve).

The QDSSC devices were fabricated by assembling the counter electrode, either gold or Cu_2_Te in nanosheet (NS), nanotube (NT), or nanoparticle (NP) form (Cu_2_Te‐NS, Cu_2_Te‐NT, Cu_2_Te‐NP, and Au), and a QD‐sensitized TiO_2_ film photo‐electrode with a binder clip separated by a 60 μm thick spacer. A metal mask with a window area of 0.16 cm^2^ was clipped onto the TiO_2_ side to define the active area of the cell when testing. The polysulfide electrolyte was composed of 2 m Na_2_S, 2 m S, and 0.2 m KCl in Milli‐Q water. For QDSSCs assembled under each set of conditions, at least six cells were prepared and tested in parallel, and average value was used as the final data. For electrochemical impedance spectroscopy (EIS) and Tafel‐polarization measurements, symmetric dummy cells were assembled from two identical CEs, using the same polysulfide electrolyte. The active area of the dummy cells was 0.64 cm^2^.^41^, ^52^

The photocurrent density–voltage (*J*–*V*) tests on the DSSCs were performed under the same sun conditions using an air mass (AM) 1.5 solar simulator (Oriel), which was carefully calibrated with certified silicon solar cells. The light intensity of the solar simulator was adjusted to 100 mW·cm^−2^ by using an optical power meter (Newport, 1918‐c) with a detector (818P‐040‐25). *J*–*V* curves were obtained by applying an external bias to the cell, and measurements were recorded by a Keithley model 2420 digital source meter. The EIS data were measured with dummy cells using a Solartron 1260 Frequency Response Analyzer in combination with a Solartron 1480 Potentiostat in the dark. The applied bias voltage and AC amplitude were set at 0 V and 10 mV, respectively. The frequency ranged from 10^6^ to 0.1 Hz. The Tafel‐polarization measurements were recorded with a scan rate of 20 mV·s^−1^ on an electrochemical workstation (CHI660d).

## Supporting information

As a service to our authors and readers, this journal provides supporting information supplied by the authors. Such materials are peer reviewed and may be re‐organized for online delivery, but are not copy‐edited or typeset. Technical support issues arising from supporting information (other than missing files) should be addressed to the authors.

Supplementary
