# Supplementary material for: Ambient Aqueous Growth of Cu2Te Nanostructures with Excellent Electrocatalytic Activity toward Sulfide Redox Shuttles
Source: Adv Sci (Weinh). 2016 Feb 3;3(5):1500350. doi: 10.1002/advs.201500350 (PMC5067604; doi:10.1002/advs.201500350)
Supplement: Supplementary file 1 — Supplementary [file ADVS-3-0n-s001.pdf]

Copyright WILEY-VCH Verlag GmbH & Co. KGaA, 69469 Weinheim, Germany, 2016.

ADVANCED SCIENCE
Open Access

# Supporting Information

for *Adv. Sci.*, DOI: 10.1002/advs.201500350

# Ambient Aqueous Growth of $Cu_2$ Te Nanostructures with Excellent Electrocatalytic Activity toward Sulfide Redox Shuttles

*Chao Han, Yang Bai, Qiao Sun, Shaohua Zhang, Zhen Li,* Lianzhou Wang,* and Shixue Dou*

Copyright WILEY-VCH Verlag GmbH & Co. KGaA, 69469 Weinheim, Germany, 2013.

# Supporting Information

**Ambient Aqueous Growth of $Cu_2Te$ Nanostructures with Excellent Electrocatalytic Activity towards Sulfide Redox Shuttles**

**Chao Han, Yang Bai, Qiao Sun, Shaohua Zhang, Zhen Li*, Lianzhou Wang*, Shixue Dou**

Chao Han, Shaohua Zhang, Prof. Zhen Li, Prof. Shixue Dou.
† Institute for Superconducting and Electronic Materials, Australian Institute for Innovative Materials, University of Wollongong, Squires Way, North Wollongong, NSW 2500, Australia.
E-mail: zhenl@uow.edu.au

Dr. Qiao Sun, Prof. Zhen Li
‡ School of Radiation Medicine and Radiation Protection, Collaborative Innovation Center of Radiation Medicine of Jiangsu Higher Education Institutions, Soochow University, 199 Ren Ai Road, Suzhou Industrial Park, Suzhou 215123, China.
E-mail: zhenli@suda.edu.cn

Dr. Yang Bai, Prof. Lianzhou Wang
§ Nanomaterials Centre, School of Chemical Engineering and Australian Institute for Bioengineering and Nanotechnology, The University of Queensland, Brisbane, QLD 4072, Australia.
E-mail: l.wang@uq.edu.cn

## 1. Tables

**Table S1**. Some physical-chemical parameters of cuprous chalcogenides.

| Material | -lg ($K_{sp}$) | JCPDS card number | Crystal structure | Molecular number in each unit cell | Volume of unit cell (Å$^3$) |
|---|---|---|---|---|---|
| $Cu_2O$ | 15 | 05-0667 | Cubic Pn-3m (224) | 2 | 77.85 |
| $Cu_2S$ | 48 | 26-1116 | Hexagonal P63/mmc(194) | 2 | 91.47 |
| $Cu_2Se$ | 61 | 06-0680 | Cubic F-43m(216) | 4 | 189.02 |
| $Cu_2Te$ | - | 10-0421 | Hexagonal P3m1(156) | 24 | 1307.13 |

**Table S2**. Reaction parameters for preparation of $Cu_2Te$ nanostructures.

| Group | Number | $Cu_2Se$:Te (molar ratio) | Size of $Cu_2Se$ (nm) | Concentration of $Na_2Te$ (mol/L) | $NaBH_4$: Te (Molar ratio) | Stirring speed (rpm) | Reaction time (min) |
|---|---|---|---|---|---|---|---|
| E1 | E1-1 | 1:1 | 11.9 | 0.01 | 80:1 | - | 2 |
| | E1-2 | 1:1 | 11.9 | 0.01 | 80:1 | - | 10 |
| | E1-3 | 1:1 | 11.9 | 0.01 | 80:1 | - | 20 |
| | E1-4 | 1:1 | 11.9 | 0.01 | 80:1 | - | 30 |
| E2 | E2-1 | 20:1 | 25.5 | 0.01 | 80:1 | - | 30 |
| | E2-2 | 10:1 | 25.5 | 0.01 | 80:1 | - | 30 |
| | E2-3 | 5:1 | 25.5 | 0.01 | 80:1 | - | 30 |
| | E2-4 | 2:1 | 25.5 | 0.01 | 80:1 | - | 30 |
| E3 | E3-1 | 1:1 | 15.0 | 0.1 | 80:1 | - | 30 |
| | E3-2 | 1:1 | 15.0 | 0.05 | 80:1 | - | 30 |
| | E3-3 | 1:1 | 15.0 | 0.02 | 80:1 | - | 30 |
| | E3-4 | 1:1 | 15.0 | 0.01 | 80:1 | - | 30 |
| E4 | E4-1 | 1:1 | 11.9 | 0.1 | 9:1 | - | 30 |
| | E4-2 | 1:1 | 15.0 | 0.1 | 9:1 | - | 30 |
| | E4-3 | 1:1 | 22.5 | 0.1 | 9:1 | - | 30 |
| | E4-4 | 1:1 | 25.5 | 0.1 | 9:1 | - | 30 |
| E5 | E5-1 | 1:1 | 22.5 | 0.1 | 9:1 | - | 30 |
| | E5-2 | 1:1 | 22.5 | 0.1 | 18:1 | - | 30 |
| | E5-3 | 1:1 | 22.5 | 0.1 | 36:1 | - | 30 |
| | E5-4 | 1:1 | 22.5 | 0.1 | 80:1 | - | 30 |
| E6 | E6-1 | 1:1 | 11.9 | 0.01 | 80:1 | 100 | 30 |
| | E6-2 | 1:1 | 11.9 | 0.01 | 80:1 | 300 | 30 |
| | E6-3 | 1:1 | 11.9 | 0.01 | 80:1 | 800 | 30 |
| | E6-4 | 1:1 | 11.9 | 0.01 | 80:1 | 1000 | 30 |

## 2. Figures.

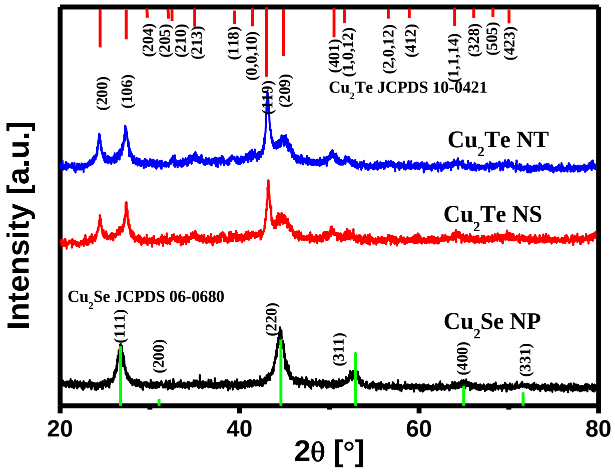


**Figure S1.** XRD patterns for $Cu_2Se$ nanoparticle precursor, and $Cu_2Te$ nanosheets and nanotubes.

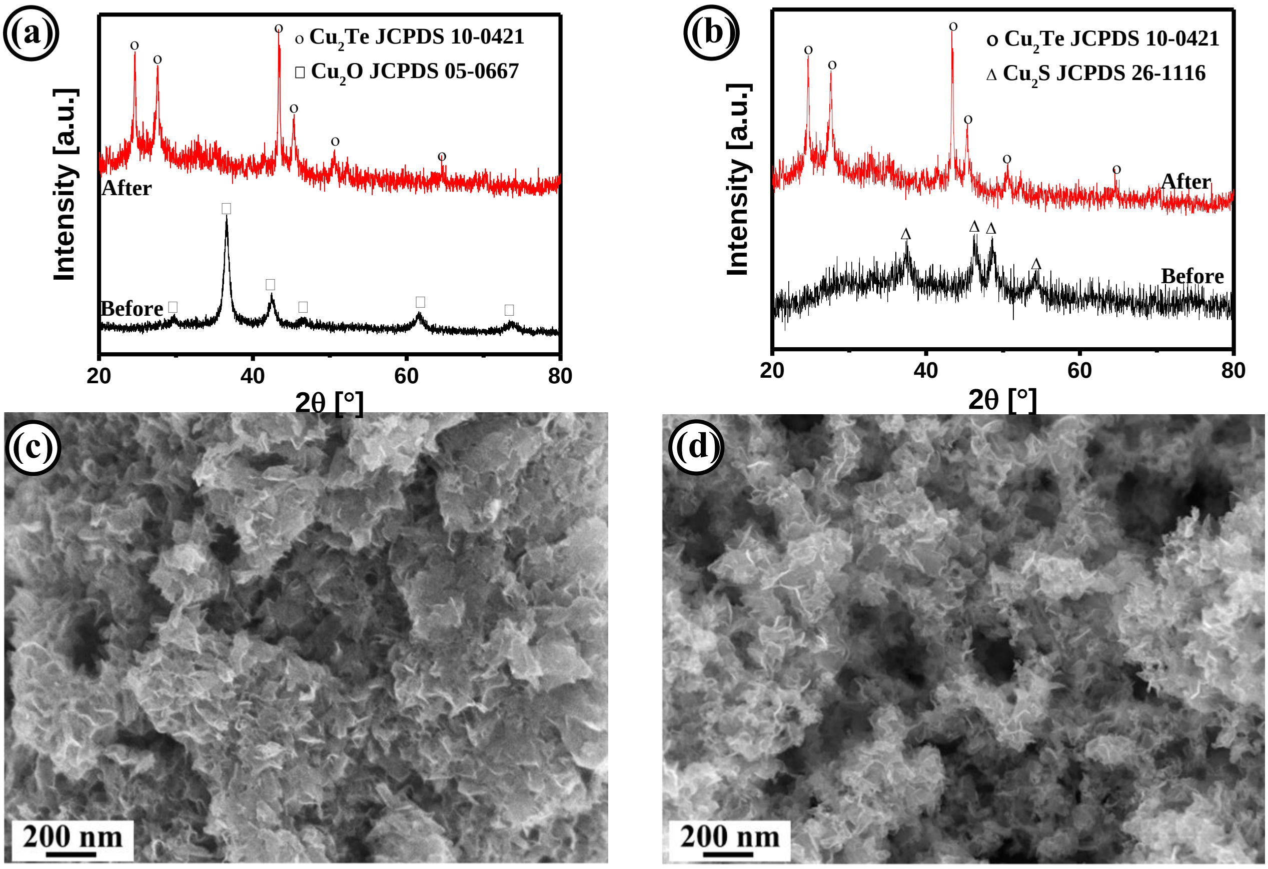


**Figure S2.** XRD patterns of $Cu_2Te$ transferred from (a) $Cu_2O$ and (b) $Cu_2S$ in comparison with that of precursors. SEM images of the $Cu_2Te$ nanosheets transferred from (c) $Cu_2O$ and (d) $Cu_2S$.

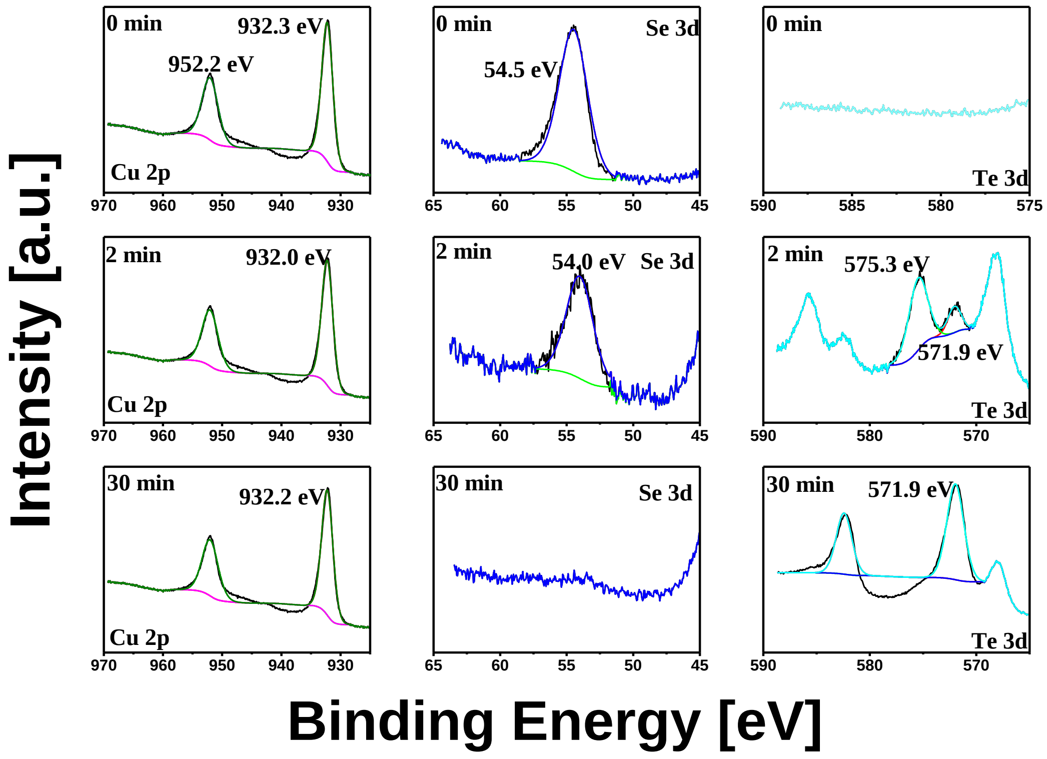


**Figure S3**. XPS spectra of $Cu_2Se$ nanoparticle precursor and the precipitates taken out after reaction of 2 min and 30 min, respectively.

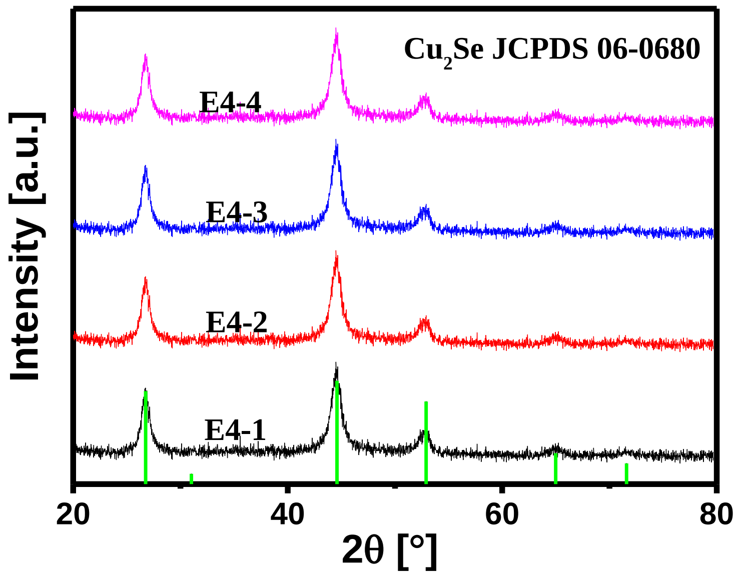


**Figure S4**. XRD patterns of $Cu_2Se$ nanoparticle precursors with different size.

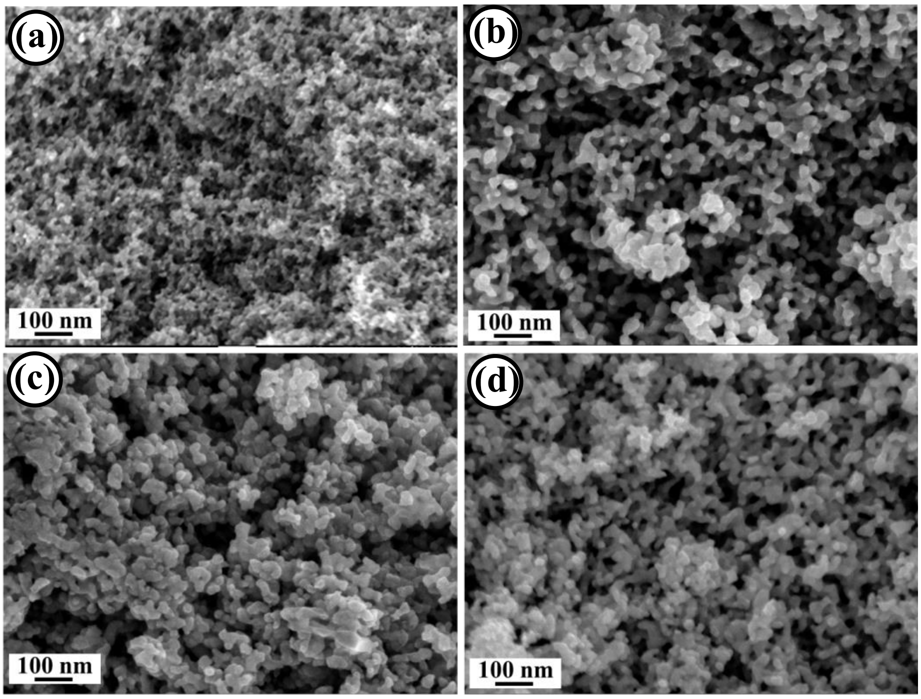


**Figure S5**. SEM images of different sized $Cu_2Se$ nanoparticle precursors used in the experiments: (a) E4-1; (b) E4-2; (c) E4-3; (d) E4-4.

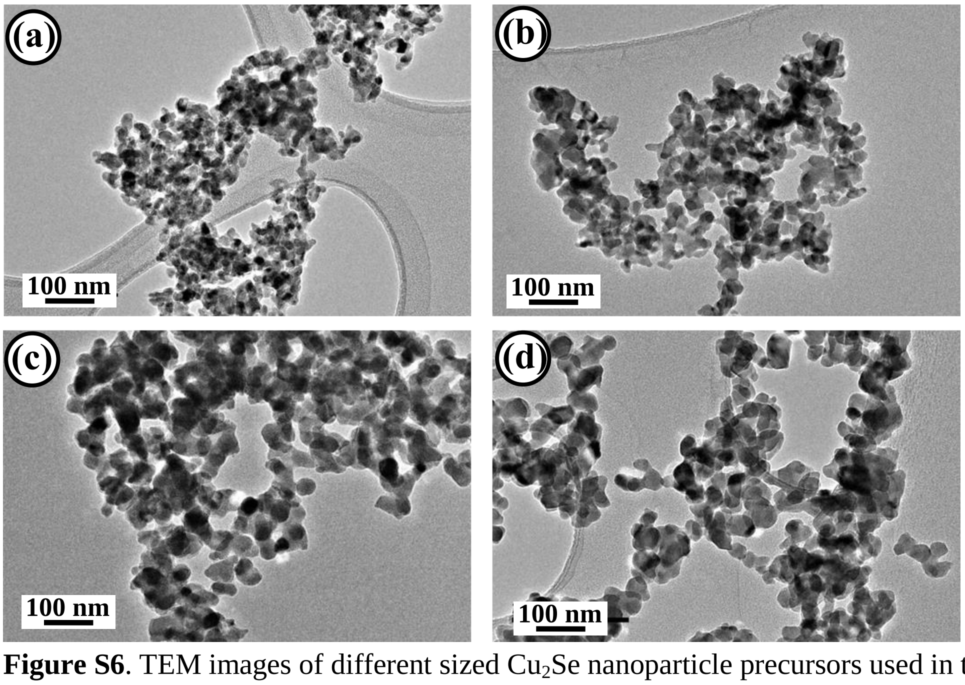


**Figure S6**. TEM images of different sized $Cu_2Se$ nanoparticle precursors used in the experiments: (a) E4-1; (b) E4-2; (c) E4-3; (d) E4-4.

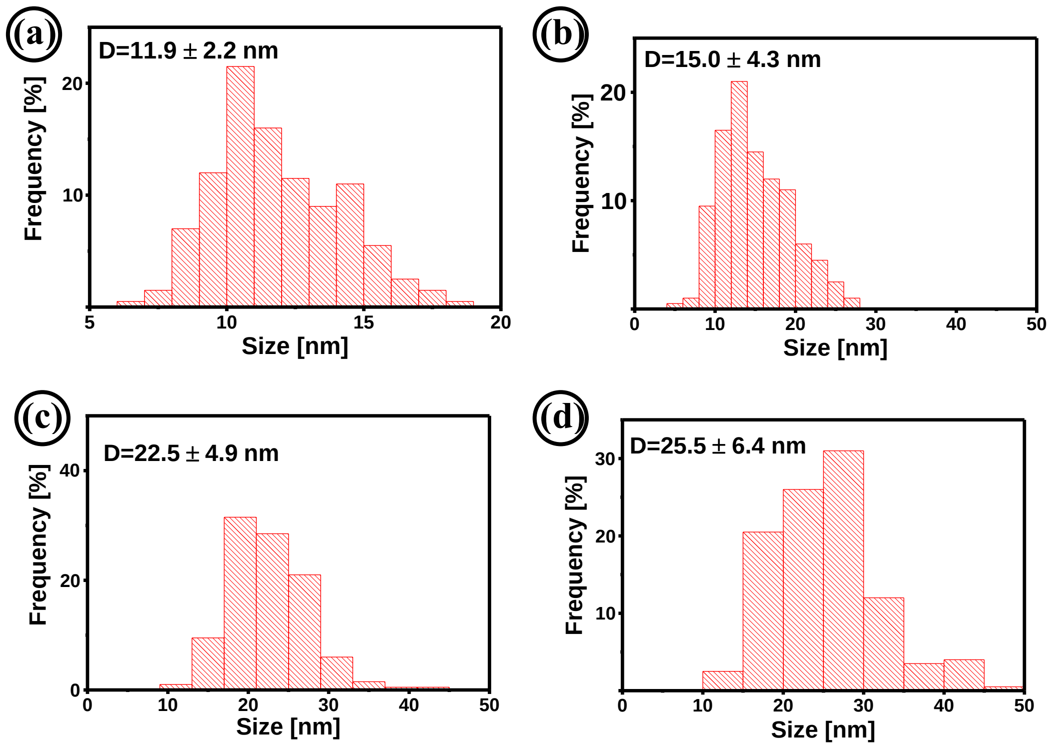


**Figure S7**. Size distributions of $Cu_2Se$ nanoparticles used in the experiments: (a) E4-1; (b) E4-2; (c) E4-3; (d) E4-4.

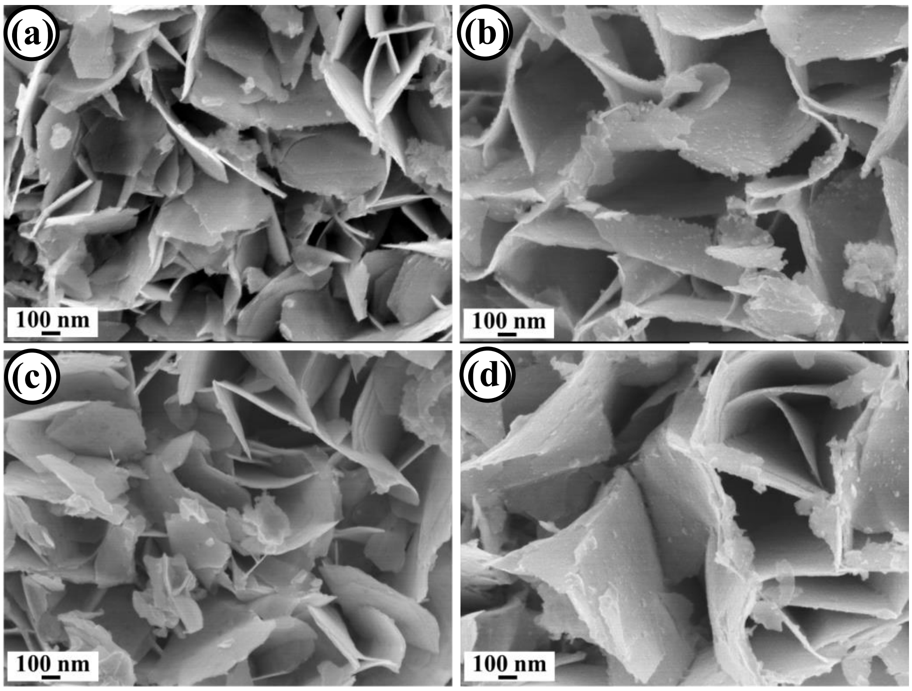


**Figure S8**. SEM images of $Cu_2Te$ nanosheets fabricated from different sized $Cu_2Se$ precursors. (a) E4-1; (b) E4-2; (c) E4-3; (d) E4-4.

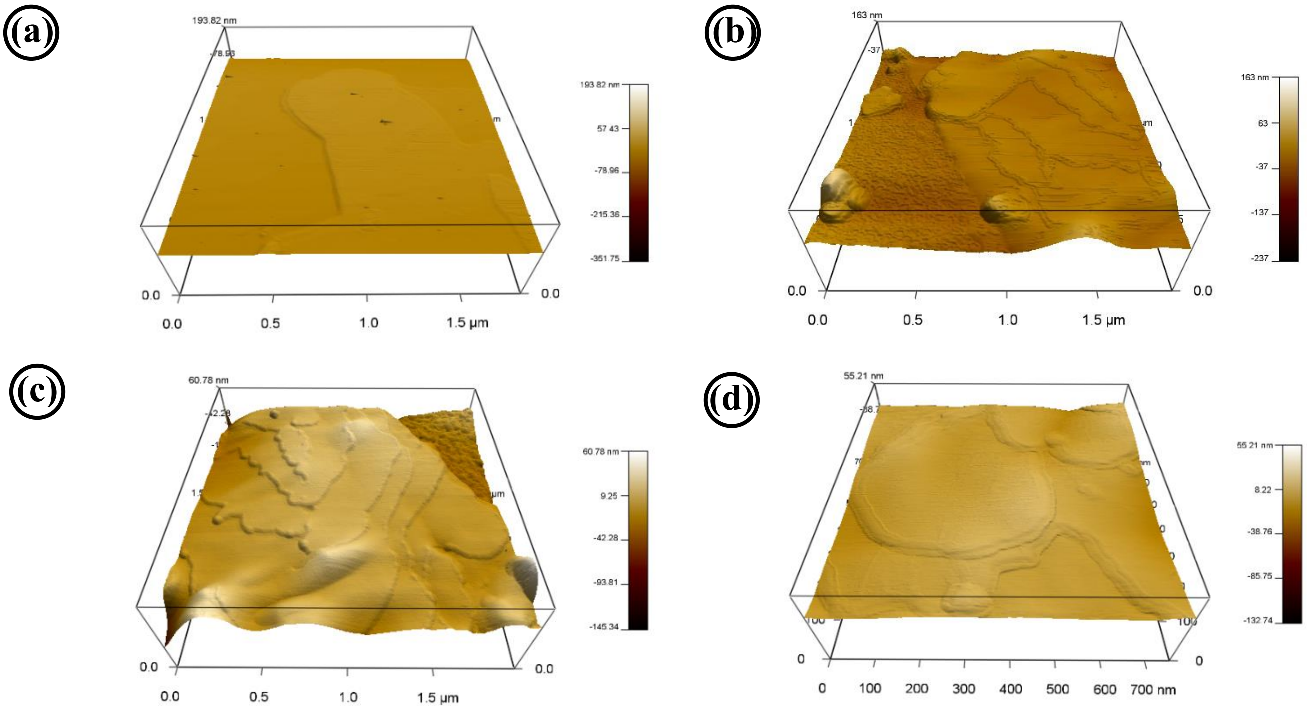


**Figure S9**. 3D AFM images of $Cu_2Te$ nanosheets fabricated from different sized $Cu_2Se$ precursors. (a) E4-1; (b) E4-2; (c) E4-3; (d) E4-4.

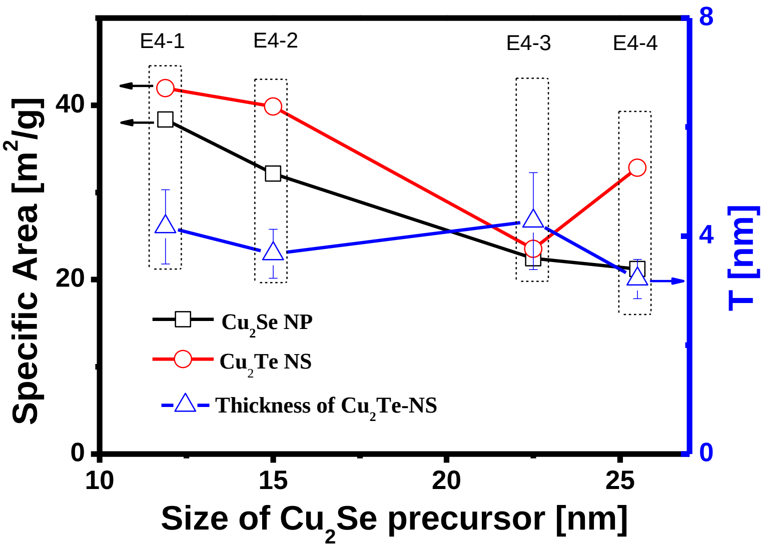


**Figure S10**. The effect of $Cu_2Se$ nanoparticle size on the specific surface area and thickness of E4-1, E4-2, E4-3 and E4-4 $Cu_2Te$ nanosheets.

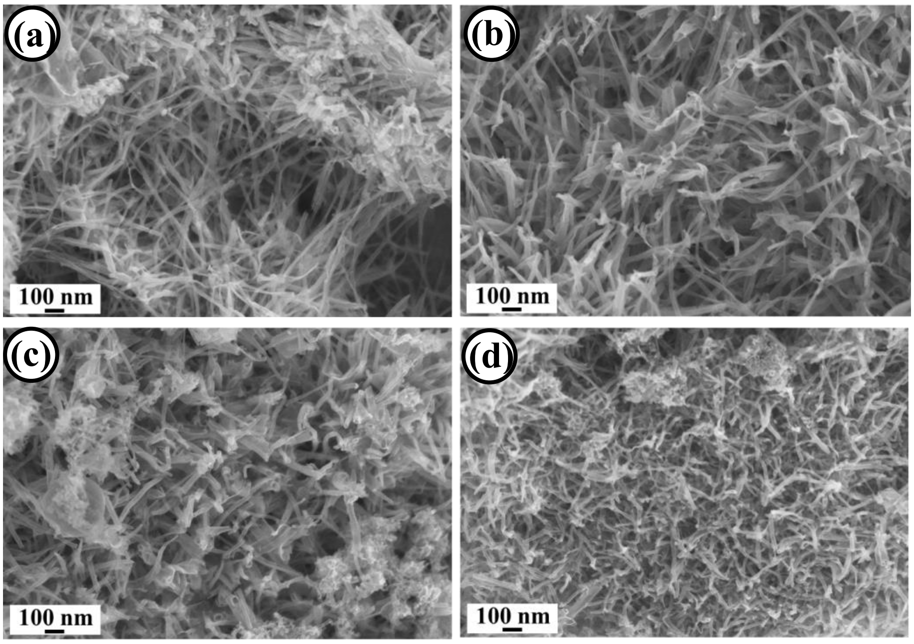


**Figure S11.** SEM images of $Cu_2Te$ nanotubes synthesized with different stirring speeds: (a) 100 rpm; (b) 300 rpm; (c) 800 rpm; (d) 1000 rpm.

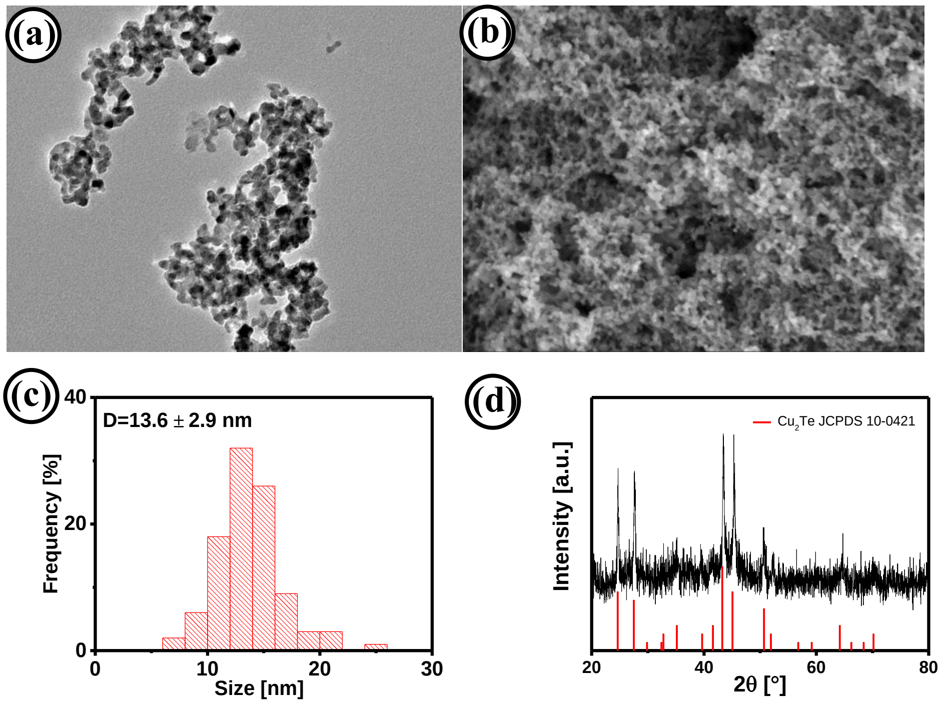


**Figure S12.** (a) TEM image; (b) SEM image; (c) particle size distribution; and (d) XRD pattern of $Cu_2Te$ nanoparticles.

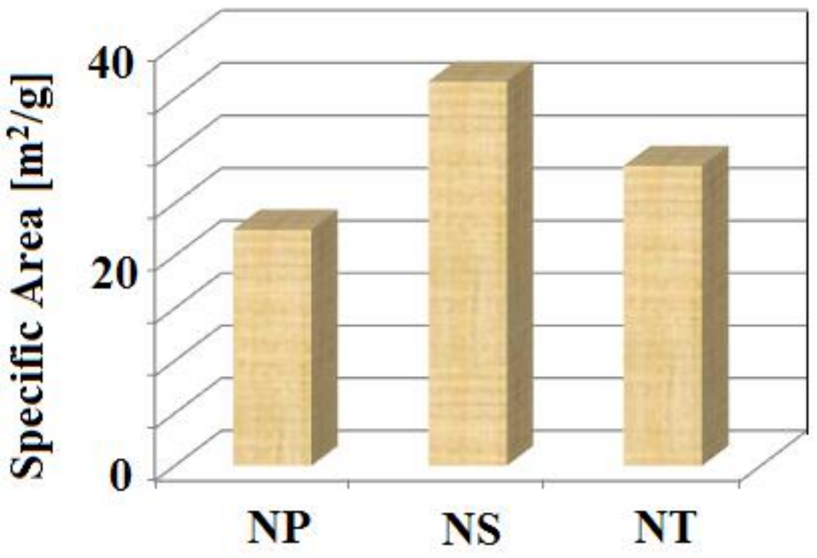


**Figure S13.** BET results of $Cu_2Te$ nanoparticles, nanosheets, and nanotubes.

### 3. Synthesis of $Cu_2Te$ nanoparticles.

1 mmol Te powder was reduced by 3 mmol $NaBH_4$ in 10 mL water to form a purple solution. Then, 10 mL, 0.2 M $CuCl_2 \cdot 2H_2O$ was added, and a black precipitate formed immediately. The product was dried in a vacuum oven after being washed with water several times.
